# Modelling the impact of infection control measures on the incidence of vancomycin-resistance enterococci: a time-series analysis

**DOI:** 10.1186/1753-6561-5-S6-P25

**Published:** 2011-06-29

**Authors:** Y-C Chen, Y-C Chuang

**Affiliations:** 1 National Taiwan University Hospital, Taipei, Taiwan, China

## Introduction / objectives

Vancomycin-resistant enterococci (VRE), an important pathogen causing healthcare associated infection emerged and increased rapidly worldwide. Several measures have been implemented to control the spread of VRE. This study aimed to evaluate the impacts of the annual hand hygiene promotion program (HHPP) and the active VRE surveillance (AS) on VRE infection/colonization incidence in a 2300-bed teaching hospital in Taiwan.

## Methods

From January 2001 through December 2010, monthly VRE infection/ colonization incidence identified according to clinical microbiology lab results and the infection control measures implemented were analyzed retrospectively. A time-series analysis was conducted using a multiple linear regression model to estimate the effects of the HHPP and the AS on the incidence of VRE infection/colonization.

## Results

A total of 1069 VRE cases were identified in 6577541 patient-days. The incidence of VRE (per 10000 patient-days) was 0.22 cases in January 2001, reached the peak of 6.40 cases in March 2009, and then decreased to 1.76 cases in December 2010. The multivariate analysis of the data showed that the incidence of VRE was significant increasing over time in these 10 years (Coefficient 0.015, P = 0.02). Implementation of the HHPP had significant decreased the incidence of VRE (Coefficient -0.599, P = 0.05). Establishment of the VRE AS also had significant decreased the incidence of VRE (Coefficient -0.971, P = 0.001). However, there was a time lag of 12-months to see the significant impact for the AS to the incidence of VRE.

## Conclusion

Though the secular trend of VRE was increasing, implementation of both hand hygiene promotion program and active VRE surveillance were effective in lowing the VRE burden. There was a time lag to the impact of active surveillance.

## Disclosure of interest

None declared.

